# Complete responders in patients with HER2-positive metastatic breast cancer: a real-world SONABRE study

**DOI:** 10.1016/j.breast.2025.104583

**Published:** 2025-09-23

**Authors:** Nan Ding, Renee Visserman, Sandra M.E. Geurts, Jolien Tol, Birgit E.P.J. Vriens, Kirsten N.A. Aaldering, Eline Boon, Marcus W. Dercksen, Franchette van den Berkmortel, Manon J.A.E. Pepels, Natascha A.J.B. Peters, Joan B. Heijns, Linda van de Winkel, Aude J.O. de Fallois, Vivianne C.G. Tjan-Heijnen, Maaike de Boer

**Affiliations:** a Division of Medical Oncology, Department of Internal Medicine, GROW School for Oncology and Reproduction, Maastricht University Medical Centre, Maastricht, the Netherlands; b Department of Internal Medicine, Jeroen Bosch Hospital, ’s Hertogenbosch, the Netherlands; c Department of Internal Medicine, Catharina Hospital, Eindhoven, the Netherlands; d Department of Internal Medicine, Laurentius Hospital, Roermond, the Netherlands; e Department of Internal Medicine, Viecuri Medical Centre, Venlo, the Netherlands; f Department of Medical Oncology, Máxima Medical Centre, Veldhoven, Eindhoven, the Netherlands; g Department of Medical Oncology, Zuyderland Medical Centre, Sittard, Heerlen, the Netherlands; h Department of Internal Medicine, Elkerliek Hospital, Helmond, the Netherlands; i Department of Internal Medicine, Sint Jans Gasthuis Hospital, Weert, the Netherlands; j Department of Medical Oncology, Amphia, Breda, the Netherlands; k Department of Internal Medicine, St Anna Hospital, Geldrop, the Netherlands

**Keywords:** Breast Neoplasms, HER-2 Gene, Pertuzumab, Disease progression, Survival

## Abstract

**Background:**

We aimed to describe real-world complete response (CR) rates, predictors of CR, and survival outcomes in patients treated with first-line pertuzumab, trastuzumab, and chemotherapy for HER2-positive (HER2+) metastatic breast cancer (MBC).

**Methods:**

Patients with HER2+ MBC who started first-line pertuzumab, trastuzumab, and chemotherapy in 2013–2021 were selected from the SONABRE Registry (NCT-03577197), involving eleven Dutch hospitals. CR was defined as no evidence of disease on imaging during first-line systemic therapy with or without local therapy (surgery and/or radiotherapy). Multivariable logistic regression was used to identify predictors for CR. Progression-free survival (PFS) and overall survival (OS) from the start of first-line pertuzumab, trastuzumab, and chemotherapy were computed with the Kaplan-Meier method.

**Results:**

We included 244 patients treated with first-line pertuzumab, trastuzumab, and chemotherapy, with a median follow-up duration of 71 months (interquartile range: 60–82 months). A CR during first-line systemic therapy was reached in 63 patients (26 %). Patients younger than 65 years (odds ratio (OR) = 5.24, 95 %CI:1.49–18.44, p = 0.01), those with *de Novo* MBC (OR = 2.48, 95 %CI:1.24–4.93, p = 0.01), or those with a single metastatic site (OR = 3.81, 95 %CI:1.83–7.96, p < 0.001) were more likely to reach CR than their counterparts. The 5-year PFS and OS rates were, respectively, 63 % (95 %CI:48 %–75 %) and 85 % (95 %CI:72 %–92 %) in patients with a CR and 10 % (95 %CI:6 %–16 %) and 36 % (95 %CI:28 %–44 %) in patients without a CR.

**Conclusions:**

In the real world, one in four patients treated with first-line pertuzumab, trastuzumab, chemotherapy, with or without local therapy, reached CR. Two third of patients with a CR were still free of progression after five years.

## Highlights

• One in four patients reached complete response on first-line HER2+ therapy.
• Younger age, *de Novo* disease, and single-site metastasis related to CR.
• Patients with CR had a 5-year OS rate of 85 % in real-world setting.

## 1 Introduction

In about one-fifth of metastatic breast cancer (MBC) patients, human epidermal growth factor receptor 2 positive (HER2+) disease is detected, representing a significant subset of the breast cancer population [1]. While metastatic HER2+ disease is generally considered incurable, a minority of patients experience a complete response (CR) [2,3]. CR refers to no evidence of disease on imaging during first-line systemic therapy with or without local therapy (surgery and/or radiotherapy) [4]. In patients receiving first-line trastuzumab-based therapy, prior real-world studies reported CR rates ranging from 10 % to 53 % [[5], [6], [7]]. In patients undergoing first-line trastuzumab-based therapy, the presence of single-organ metastasis, premenopausal status, *de Novo* metastases, and surgery of the primary tumour or a metastatic lesion have been identified as predictive factors for achieving CR [8,9]. Patients with HER2+ MBC who attained a CR on trastuzumab-based chemotherapy regimens may be potential candidates for long-term survivorship [[7], [8], [9]].

The landmark CLEOPATRA trial revealed that integrating pertuzumab into the standard trastuzumab and docetaxel regimen markedly improved overall survival (OS) in HER2+ MBC patients, extending it from a median of 41–57 months [10]. Notably, 68 % of patients receiving the combination of trastuzumab, pertuzumab, and docetaxel achieved either a partial or complete response, surpassing the 57 % response rate observed in the trastuzumab-docetaxel treatment group.

This real-world study aimed to explore the CR rates, predictors for CR, and progression-free survival (PFS) and OS in patients with HER2+ MBC treated with first-line pertuzumab, trastuzumab, and chemotherapy.

## 2 Patients and methods

### 2.1 SONABRE registry

The SOutheast Netherlands Advanced BREast cancer (SONABRE) Registry (NCT-03577197) is an ongoing cohort study that aims to include all patients aged 18 years or older who have been diagnosed with MBC in the Southeast of the Netherlands since 2007. Data were extracted from electronic health records by trained registry clerks. They collect patient and tumour characteristics, as well as treatment information, response, progression and death. The SONABRE registry is governed by the Department of Medical Oncology at MUMC+. Data ownership resides with the registry committee. De-identified datasets are available to participating institutions upon reasonable request, subject to data-sharing agreements and ethical approvals. Since SONABRE includes consecutive patients from academic, teaching, and general hospitals, selection bias is limited.

For the current study, we selected all patients with HER2+ MBC who received pertuzumab, trastuzumab, and chemotherapy as first-line systemic therapy between 2013 and 2021 in 11 hospitals. Endocrine maintenance therapy in patients with hormone-receptor positive disease was allowed. The last follow-up was collected in 2023 or 2024, depending on the hospital. The data lock was on October 7th, 2024.

### 2.2 Definitions

HER2 positivity was defined as either an immunohistochemistry (IHC) score of 3+ or an IHC score of 2+ combined with a positive in situ hybridisation (ISH) result, assessed on a metastatic lesion or, if not available, on the primary tumour. Hormone receptor positivity was determined by a nuclear staining percentage of at least 10 % for oestrogen and/or progesterone receptors. *De Novo* metastatic disease was defined as the radiologic and/or pathologic diagnosis of at least one metastatic lesion within three months after the diagnosis of the primary breast cancer.

The treating physician assessed the clinical response to first-line treatment based on imaging, tumour markers, and clinical signs and symptoms. This was registered by registration clerks as CR, partial response, stable disease, or progressive disease [8,11]. Objective response included complete response and partial response. Radiological response was evaluated by treating physicians according to daily practice, often informed by RECIST criteria, although formal RECIST measurement was not systematically applied. In this study, we defined CR as no evidence of disease on imaging during first-line pertuzumab, trastuzumab, and taxane-based chemotherapy, which may have included the use of radiotherapy and surgery of the primary tumour or metastases to achieve a complete response. Imaging modalities used for assessment included FDG-positron emission tomography (PET)-CT, bone scintigraphy, or CT scan. Bone lesions were considered to have completely responded when residual sclerotic lesions on CT had become metabolically inactive on PET-CT or resolved on bone scintigraphy. Information bias may have occurred due to variation in imaging schedules and clinical documentation, reflecting the nature of real-world practice. The body mass index (BMI) was calculated from weight and height (BMI = weight (kg)/height (m)^2^), measured by the treating physician or self-reported by the patient at diagnosis. In accordance with the World Health Organization criteria, BMI was categorised as underweight (<18.5 kg/m^2^), normal weight (18.5–24.9 kg/m^2^), overweight (25.0–29.9 kg/m^2^), or obese (≥30.0 kg/m^2^).

### 2.3 Endpoints and statistical analyses

Patient and tumour characteristics, along with details of prior (neo)adjuvant therapy, local therapy, and first-line palliative chemotherapy, were analysed descriptively for all patients, those with and without CR, treated with first-line pertuzumab, trastuzumab, and chemotherapy. Patient and tumour characteristics included age at the start of first-line systemic therapy (<65 years and ≥65 years), comorbidity (any and cardiovascular), WHO performance status (0–1, 2, and unknown), BMI, hormone receptor status (positive and negative), metastatic-free interval (<3 months/*de Novo*, 3–23 months, and ≥24 months), number of metastatic sites (single and multiple), metastatic site (bone, lymph node and soft tissue, visceral, and central nervous system (CNS)), prior (neo)adjuvant systemic therapy (among patients with metachronous metastases: any, chemotherapy, and trastuzumab), chemotherapeutic backbone of first line palliative therapy (anthracycline and taxane, taxane without anthracycline, and other), and local therapy (radiotherapy of metastases, surgery of metastases, and surgery of the primary tumour in patients with *de Novo* metastases). Objective responses were summarised using descriptive statistics.

Time from the start of first-line pertuzumab, trastuzumab, and chemotherapy to the date of CR was estimated using the competing risk method. CR was the event of interest, while progression, the start of a new line of therapy (excluding endocrine maintenance), or death were considered competing events. Patients who had not reached CR on first-line treatment at last follow-up, nor experienced a competing event, were censored.

To identify factors associated with CR, logistic regression was performed. Variables included age, BMI, hormone receptor status, metastatic-free interval, number of metastatic sites, and metastatic site. Factors with a p-value <0.20 in the univariable analysis were included in the multivariable model. Residual confounding may be present as some prognostic factors (e.g. palliative radiotherapy and oligo-metastases) were not consistently available [7,8].

PFS was defined as the time from the start of first-line pertuzumab, trastuzumab, and chemotherapy to disease progression or death, and was censored at the start of a next-line treatment initiated for reasons other than progressive disease. OS was defined as the time from the start of first-line pertuzumab, trastuzumab, and chemotherapy to the date of death. If no PFS or OS event occurred, patients were censored at the date of last follow-up.

We analysed median and five-year PFS and OS outcomes in patients who reached a CR and those who did not. PFS and OS were computed with the Kaplan-Meier method.

Statistical analyses were conducted with SPSS version 28.0 (IBM Corp., Armonk, NY, USA) and Stata version 17.0 (StataCorp., College Station, Texas, USA). Figures were generated using Stata version 17.0 (StataCorp., College Station, Texas, USA). A two-tailed p-value of less than 0.05 was regarded as statistically significant. This study followed the ESMO guidance for reporting oncology real-world evidence [12].

## 3 Results

**Fig. 1:**
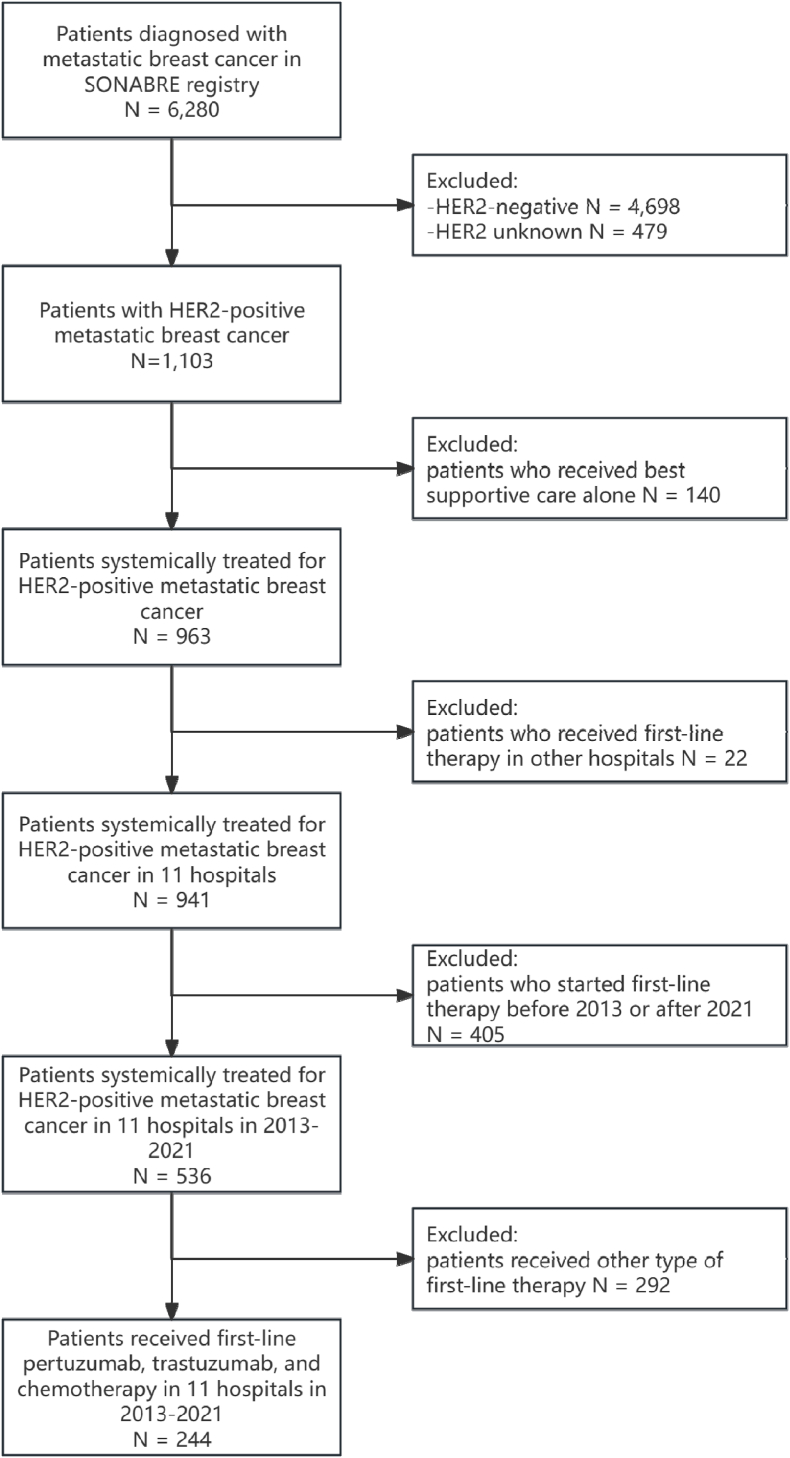
Flow chart HER2 = human epidermal growth factor receptor 2, N = number of patients, SONABRE= SOutheast Netherlands Advanced BREast cancer.

Among the 6280 patients with MBC included in the SONABRE registry, 1103 were diagnosed with HER2+ MBC. Among them, 244 patients received first-line pertuzumab, trastuzumab, and chemotherapy in 11 hospitals in 2013–2021 and were included in the present analyses (Fig. 1). Among them, 83 % were aged under 65 years, and 91 % had a WHO performance status of 0–1 and 41 % had at least one comorbidity (Table 1). The tumour was hormone receptor positive in 53 % of patients, 62 % had multiple metastatic sites, and 69 % had visceral metastases. Fifty-three percent of the patients had *de Novo* MBC. Among patients with metachronous MBC (n = 115), 73 % had previously received chemotherapy, and 57 % had previously received trastuzumab-based therapy in the neoadjuvant or adjuvant setting. Among all patients (n = 244), the chemotherapy backbone of the first-line palliative pertuzumab, trastuzumab, and chemotherapy regimen was taxane without anthracycline in 94 % of cases, antracycline and taxane in 5 % of cases, and other chemotherapy (vinorelbine, carboplatin) in 1 % of cases. Surgery of metastatic site(s) was performed in 12 % of all patients, and radiotherapy of metastatic site(s) was performed in 46 % of all patients. Among patients with *de Novo* MBC (n = 129), 36 % underwent surgery of the primary tumour.

**Table 1:** Patient, tumour, and treatment characteristics of the 244 patients with HER2+ MBC receiving first-line pertuzumab, trastuzumab and chemotherapy, and for the 63 patients with complete response and 181 without, N (%).

Characteristic	Category	All patients N = 244 (%)	Patients with CR N = 63 (%)	Patients without CR N = 181 (%)
*Patient*				
Age at start of treatment	<65 years	202 (83)	60 (95)	142 (79)
	65+ years	42 (17)	3 (5)	39 (22)
Comorbidity	Any	101 (41)	25 (40)	76 (42)
	Cardiovascular	45 (18)	6 (10)	39 (22)
WHO-performance status	0–1	213 (91)	58 (98)	155 (89)
	2	21 (9)	1 (2)	20 (11)
	Unknown	N = 10	N = 4	N = 6
**BMI**	Underweight	4 (2)	N = 0	4 (2)
	Normal	108 (46)	32 (51)	76 (44)
	Overweight	75 (32)	20 (32)	55 (32)
	Obese	49 (21)	11 (18)	38 (22)
	Unknown	N = 8	N = 0	N = 8
*Tumour*				
Hormone receptor status	Positive	128 (53)	31 (49)	97 (54)
	Negative	116 (48)	32 (51)	84 (46)
Metastatic-free interval	<3 months/*de Novo*	129 (53)	47(75)	82 (45)
	3–23 months	15 (6)	2 (3)	13 (7)
	≥24 months	100 (41)	14 (22)	86 (48)
Number of organs involved	Single	92 (38)	39 (62)	53 (29)
	Multiple	152 (62)	24 (38)	128 (71)
Metastatic site	Bone	150 (62)	26 (41)	124 (69)
	Lymph node and Soft tissue^a^	125 (51)	26 (41)	99 (55)
	Visceral^b^	169 (69)	38 (60)	131 (72)
	CNS^c^	19 (8)	1 (2)	18 (10)
*Treatment*				
(Neo)adjuvant chemo and/or HER2-targeted therapy in patients with metachronous metastases (i.e. MFI >3 months)	Any	91 (79)	12 (75)	79 (80)
Chemotherapeutic backbone of first line palliative therapy	Chemotherapy	84 (73)	12 (75)	72 (73)
	Trastuzumab	66 (57)	8 (50)	58 (59)
	Anthracycline and taxane	11 (5)	7 (11)	4 (2)
	Taxane without anthracycline	230 (94)	56 (89)	174 (96)
	Other	3 (1)^d^	N = 0	3 (2)^d^
*Local therapy*				
Radiotherapy of metastases	Yes	112 (46)	32 (51)	80 (44)
Surgery of metastases	Yes	29 (12)	10 (16)	19 (11)
Surgery of the primary tumour in patients with *de Novo* metastases	Yes	46 (36)	31 (66)	15 (18)
	No	82 (64)	16 (34)	67 (82)

BMI = body mass index, CR = complete response, CNS = central nervous system, HER2 = human epidermal growth factor receptor 2, N = number of patients, NA = not applicable, MBC = metastatic breast cancer, MFI = Metastatic-free interval, WHO= World Health Organization.

a Lymph nodes and skin.

b Liver, lung, pleura, peritoneum, gastrointestinal track, kidney, adrenal and ovaries.

c Brain, leptomeningeal, and eye.

d Other included vinorelbine (n = 2) and carboplatin (n = 1).

### 3.1 Complete response rate and predictors of complete response

**Fig. 2:**
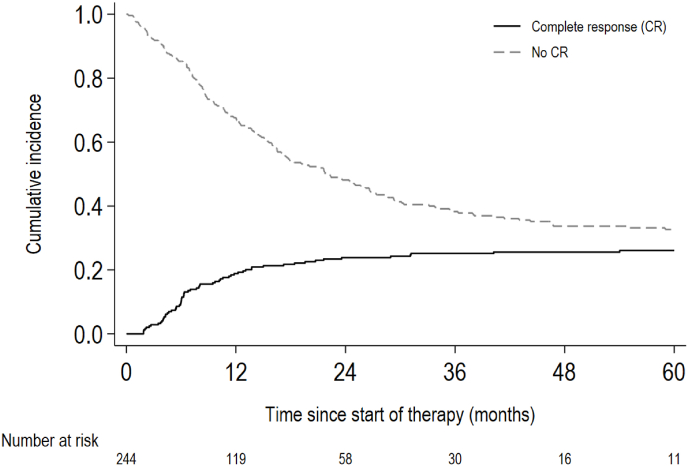
Time to complete response in patients treated with first-line pertuzumab, trastuzumab and chemotherapy *The ‘number at risk’ and the area between the curves represent respectively the number and the proportion of patients progression-free and without prior defined complete response*.

The median follow-up duration of the study population was 71 months (interquartile range: 60–82 months). An objective response as best response to first-line pertuzumab, trastuzumab, and chemotherapy was observed in 207 patients (85 %) (Supplementary Table 1). Sixty-three patients had a CR on first-line pertuzumab, trastuzumab, and chemotherapy, with a five-year CR rate after initiating first-line systemic therapy of 26 % (95 % confidence interval (CI): 21 %–32 %) (Fig. 2). Among the 63 patients who reached CR, 57 (90 %) reached CR on all lesions after systemic therapy (Supplementary Fig. 1). Of these, 23 reached CR only on systemic therapy. Twelve patients (19 %) reached CR through a concurrent combination of systemic therapy and local therapy of the primary tumour and/or metastases. Another 22 patients reached CR on systemic therapy and received additional local therapy afterwards. Six patients (10 %) did not reach a CR on all lesions with first-line systemic therapy, and needed local therapy to reach a CR. A case example per scenario is presented in **Supplementary Text 1**.

In multivariable analysis, the odds of obtaining a CR was statistically significantly higher in patients younger than 65 years compared with older patients (adjusted OR = 5.24, 95 % CI: 1.49–18.44, p = 0.01), in those with *de Novo* MBC compared with metachronous MBC (adjusted OR = 2.48, 95 % CI: 1.24–4.93, p = 0.01), and in those with a single metastatic site compared with multiple metastatic sites (adjusted OR = 3.81, 95 % CI: 1.83–7.96, p < 0.001) (Table 2).

**Table 2:** Predictors for complete response to first-line pertuzumab, trastuzumab and chemotherapy.

Characteristic	N	% with CR	Univariable OR (95 % CI)	Multivariable OR (95 % CI)
**Age at start of treatment**				
<65 years	202	30 %	5.49 (1.63–18.57), p = 0.006	5.24 (1.49–18.44), p = 0.01
65+ years	42	7 %	Ref.	Ref.
**BMI**				
Underweight	4	0	–	–
Normal	108	30 %	Ref.	–
Overweight	75	27 %	0.86 (0.45–1.67), p = 0.66	–
Obese	49	22 %	0.69 (0.31–1.51), p = 0.69	–
**Hormone receptor status**				
Positive	128	25 %	0.84 (0.47–1.49), p = 0.55	–
Negative	116	28 %	Ref.	–
**Metastatic-free interval**				
<3 months/*de Novo*	129	37 %	3.55 (1.87–6.72), p < 0.001	2.48 (1.24–4.93), p = 0.01
≥3 months	115	14 %	Ref.	Ref.
**Number of metastatic sites**				
Single	92	43 %	3.93 (2.15–7.16), p < 0.001	3.81 (1.83–7.96), p < 0.001
Multiple	152	16 %	Ref.	Ref.
**Metastatic site**				
Bone only	37	38 %	1.79 (0.83–3.85), p = 0.14	0.70 (0.28–1.79), p = 0.70
Lymph node and soft tissue^a^	29	41 %	2.33 (1.02–5.31), p = 0.05	2.41 (0.96–6.07), p = 0.06
Visceral^b^	159	23 %	Ref.	Ref.
CNS	19	5 %	0.18 (0.02–1.42), p = 0.10	0.16 (0.02–1.38), p = 0.10

BMI = body mass index, CR = complete response, N = number of patients, OR = odds ratio, Ref. = reference, CNS= Central nervous system.

a Without visceral or CNS.

b Wihout CNS.

### 3.2 Survival outcomes

**Fig. 3:**
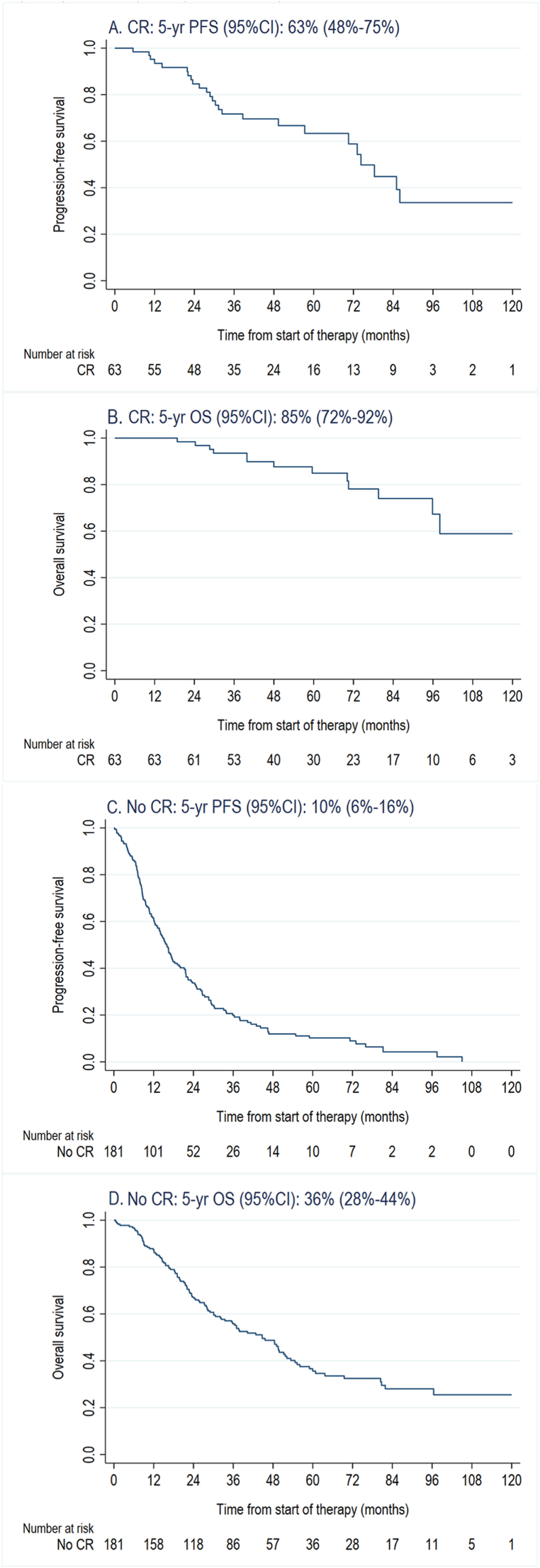
Progression-free survival (PFS, A and C) and overall survival (OS, B and D) from the start of pertuzumab, trastuzumab and chemotherapy stratified by reaching complete response (CR, A and B) or not (no CR, C and D).

Patients who achieved a CR on first-line pertuzumab, trastuzumab, and chemotherapy had a median first-line PFS of 74.3 months (95 % CI: 57.3-not reached (NR) at 122.0 months) and a five-year PFS rate of 63 % (95 % CI: 48 %–75 %) (Fig. 3A). For these patients, the median OS was not reached at 122.0 months of follow-up (95 % CI: 95.9-NR), with a five-year OS rate of 85 % (95 % CI: 72 %–92 %) (Fig. 3C). Patients who did not achieve a CR on first-line pertuzumab, trastuzumab, and chemotherapy had a median PFS of 16.0 months (95 % CI: 13.0–18.4) and a five-year PFS rate of 10 % (95 % CI: 6 %–16 %) (Fig. 3B). The median OS for this group was 44.7 months (95 % CI: 33.5–51.7), and the five-year OS rate was 36 % (95 % CI: 28 %–44 %) (Fig. 3D).

Among all 244 patients, the median PFS on first-line pertuzumab, trastuzumab, and chemotherapy was 22.3 months (95 % CI: 17.7–26.6), with a five-year PFS rate of 24 % (95 % CI: 18 %–30 %) (Supplementary Fig. 2A). The median OS was 59.6 months (95 % CI: 49.6–80.5), and the five-year OS rate was 49 % (95 % CI: 41 %–55 %) (Supplementary Fig. 2B).

## 4 Discussion

In this real-world study of HER2+ MBC patients treated with first-line pertuzumab, trastuzumab, and chemotherapy between 2013 and 2021, 26 % of patients achieved a CR. Patients with an age below 65 years, *de Novo* MBC, or a single metastatic site had a two-to five-fold higher likelihood of obtaining a CR. Patients reaching a CR had a favourable prognosis with a 5-year first-line PFS of 63 %. To our knowledge, this is the first study to describe CR rates, predictors of CR and prognosis in a real-world population treated with first-line pertuzumab added to chemotherapy and trastuzumab.

The majority of patients reached the CR within one year of the start of first-line treatment. In our study, CR was defined by the treating physician in daily clinical practice, where imaging is performed less often than in clinical trials. The true moment of reaching CR may therefore be earlier than reported in our study. Prior studies reported varying CR rates in patients on HER2-targeted treatment. For example, in the CLEOPATRA trial, a 13 % CR rate was reported in patients receiving pertuzumab, trastuzumab, and chemotherapy, but they only considered CR at 9 weeks of follow-up [13]. Furthermore, our CR rate also exceeds those reported in the DESTINY-Breast09 trial, in which CRs were observed in 15 % of patients treated with trastuzumab deruxtecan (T-DXd) and pertuzumab, and 9 % of those treated with pertuzumab, trastuzumab and chemotherapy, although the protocol has not publicly specified the assessment time point [16]. Others observational studies reported CR rates of 10–21 % in cohorts of patients in which 3–33 % of patients received pertuzumab [7,8,14]. Veitch et al. and Kaplan et al. found higher CR rates (45 % and 54 %, respectively) in their observational cohorts due to the selection of long-term responders in which 51 % had used pertuzumab [9,15]. The lack of clarity on the timing of response assessments may partly explain differences in reported CR rates between trials and real-world practice. In contrast to our study, where CR could be reached after systemic therapy with or without surgery, surgical interventions were not part of the CLEOPATRA and DESTINY-Breast09 protocol [13.16]. Given the higher CR rate observed in the T-DXd arm in DESTINY-Breast09, CR rates in real-world practice are expected to be even higher than we have reported now, once T-DXd becomes available in the first-line setting in the Netherlands. The variability in CR rates is likely to be explained by differences in patient selection, given therapies, and response definitions and evaluations.

We observed higher CR rates in three specific patient groups: those younger than 65 years old (compared with older patients), those with *de Novo* MBC (compared with metachronous MBC), and those with single-organ metastases (compared with multiple-organ metastases). Patients under 65 years were approximately five times more likely to achieve a CR compared with older patients. Potential reasons for higher CR rates could be explained by higher likelihood of radiotherapy and surgery and better toleration of aggressive treatments in younger patients [[17], [18], [19]]. However, Wong et al. reported no association between age at diagnosis and achieving CR in patients with *de novo* HER2+ MBC (>50 years vs. ≤50 years: adjusted OR = 0.95, 95 % CI: 0.53–1.71, p = 0.87) [8]. This discrepancy may stem from differences in age categorisation and patient populations between the studies. Patients with single-organ metastases were 3.5 times more likely to achieve CR than those with multiple-organ metastases. In line with our findings, Wong et al. showed that patients with two metastatic sites had significantly lower CR rates than those with a single metastasis [8]. In contrast, Veitch et al. found no association between the number of organs involved and achieving CR among long-term responders [9]. Patients with single-organ metastases usually present with a lower tumour burden. As a result, they are more likely to receive local therapy of the primary tumour or metastases, which could contribute to the improved CR rate [[20], [21], [22]]. Steenbruggen et al. found that patients with only one metastasis more often received local therapy of the primary tumour than those with multiple sites (54 % vs. 34 %) [23]. Although the use of additional local therapy may be associated with CR, it is important to note that only 6 patients (10 %) in our cohort reached CR only through local therapy. Patients with *de Novo* HER2+ MBC were 2.5 times more likely to achieve CR compared to those with metachronous metastases. This aligns with Veitch et al., who demonstrated that *de Novo* MBC was associated with a fivefold higher likelihood of achieving CR [9]. Provided the fact that these patients are treatment naïve, patients with *de Novo* MBC are more sensitive to HER2-targeted therapy, including trastuzumab and pertuzumab, which can explain the increased CR rates [[24], [25], [26]].

The proportion of patients undergoing surgery of the primary tumour (36 % of those with *de Novo* MBC) in our study was relatively high compared with the 29 % reported in a Dutch nationwide study focusing on patients with *de Novo* HER2+ MBC patients and also higher than the 15–27 % reported in other countries [20,21,27,28]. The difference between our SONABRE cohort and other cohorts may partly reflect variations in clinical practice and institutional policy, due to the lack of consistent evidence of therapeutic benefit. Although randomized trials such as ECOG-ACRIN E2108 and MF07-01 failed to demonstrate a survival advantage in unselected populations, some retrospective studies have suggested that surgery of the primary tumour may improve outcomes in carefully selected patients [20,21,27,29,30]. It remains uncertain whether surgery contributes directly to improved CR rates or OS or whether the observed outcomes are primarily driven by patient selection. Future studies are needed to clarify the role of loco-regional therapy in the metastatic setting and to identify subgroups of patients who may or may not benefit from surgical intervention.

Patients who achieved a CR in our study had an excellent prognosis, consistent with previous literature linking CR to prolonged PFS and OS [[31], [32], [33]]. Veitch et al. reported outcomes for long responders, defined as patients whose response duration to first-line trastuzumab and chemotherapy or pertuzumab, trastuzumab, and taxane was at least twice as long as observed in clinical trial [9]. Among these long responders, the 5-year PFS and OS rates were 93.2 % and 97.4 %, respectively. Wong et al., including only patients with *de Novo* HER2+ MBC, reported 5-year PFS rates of 100 % and 5-year OS rates of 98 % among patients with CR, and these remained the same at 10 years of follow-up [8]. In contrast, patients who did not reach CR in their study had significantly lower 5-year PFS (11 %) and OS (61 %) rates. Our unselected cohort was more heterogeneous than those of Veitch and Wong, which may explain the lower but still favourable OS rates observed in our study. Steenbruggen et al. showed a median and 5-year OS of 142 months and 52 %, compared with 35 months and 7 % for patients without CR in patients receiving trastuzumab and chemotherapy [7]. These lower rates compared to our findings might be attributable to differences in study populations or methodological aspects, rather than treatment era alone.

Notably, only 25 % of the patients with HER2+ MBC treated in the 11 hospitals in the southeast of the Netherlands received first-line pertuzumab, trastuzumab, and chemotherapy. This may be explained by the fact that pertuzumab was only reimbursed in the Netherlands from 2013 onward, with uptake varying by hospital and year [34]. Furthermore, some patients were ineligible for intensive combination therapy due to comorbidities or poor performance status. This study represents a comprehensive, multi-centre, real-world analysis of data from eleven Dutch hospitals, including academic, teaching, and general hospitals. A notable strength of this study is its long-term follow-up period and detailed data collection on variables influencing CR, with particular value in including patients achieving CR in cases of bone metastases, which is a group often excluded in clinical trials using RECIST 1.1 criteria. In this observational study, CR was defined by the treating physician using different imaging modalities according to local practice such as PET-CT scans, bone scans, and CT scans. Of relevance, we included patients who reached CR following local therapy, either alone or in combination with systemic therapy, reflecting real-world clinical practice. As this is a retrospective study, limitations include non-standardized imaging and treatment decisions, as well as potential residual confounding despite multivariable adjustment. Due to the immortal time bias, PFS and OS are inherently longer in patients with CR compared with those without. As a result, we chose not to directly compare survival outcomes between the CR and non-CR groups.

The clinical implications of achieving CR in patients with HER2+ MBC remain uncertain. CR may represent a prognostic marker for excellent long-term outcomes, but whether it can be used to guide treatment de-escalation or, conversely, to support continuation of the same therapy requires prospective evaluation. Similarly, it is unclear whether patients with only stable or partial responses should continue current therapy until progression, or whether earlier treatment escalation might improve outcomes. These questions cannot be answered from our data but highlight important avenues for future research, including whether patients who remain progression-free for more than five years can be considered functionally cured and whether maintenance endocrine therapy contributes to sustaining such long-term responses.

## 5 Conclusion

In this real-world study of 244 patients with HER2+ MBC treated with first-line pertuzumab, trastuzumab, and chemotherapy, one in four patients achieved a CR, which was associated with a favourable prognosis.

## CRediT authorship contribution statement

**Nan Ding:** Writing – review & editing, Writing – original draft, Visualization, Validation, Methodology, Investigation, Formal analysis, Data curation, Conceptualization. **Renee Visserman:** Writing – review & editing. **Sandra M.E. Geurts:** Writing – review & editing, Visualization, Validation, Supervision, Methodology, Investigation, Formal analysis, Data curation. **Jolien Tol:** Writing – review & editing, Resources. **Birgit E.P.J. Vriens:** Writing – review & editing, Resources. **Kirsten N.A. Aaldering:** Writing – review & editing, Resources. **Eline Boon:** Writing – review & editing, Resources. **Marcus W. Dercksen:** Writing – review & editing, Resources. **Franchette van den Berkmortel:** Writing – review & editing, Resources. **Manon J.A.E. Pepels:** Writing – review & editing, Resources. **Natascha A.J.B. Peters:** Writing – review & editing, Resources. **Joan B. Heijns:** Writing – review & editing, Resources. **Linda van de Winkel:** Writing – review & editing, Resources. **Aude J.O. de Fallois:** Writing – review & editing, Resources, Conceptualization. **Vivianne C.G. Tjan-Heijnen:** Writing – review & editing, Supervision, Methodology, Conceptualization. **Maaike de Boer:** Writing – review & editing, Validation, Supervision, Resources, Methodology, Investigation, Conceptualization.

## Ethics approval

The registry is authorised by the board of directors of the Academic Hospital Maastricht and the Medical Research Ethics Committee of the Maastricht University Medical Centre (15-4-239).

## Funding information

The SONABRE Registry was supported by the Netherlands Organization for Health Research and Development (ZonMw: 80–82500-98–8003, 10140312310018); Novartis BV; Roche; Pfizer; Eli Lilly & Co; Gilead and AstraZeneca. Funding sources had no role in the conceptualisation or writing of the manuscript.

## Declaration of competing interest

The authors declare the following financial interests/personal relationships which may be considered as potential competing interests:DN reports grants scholarship from the China Scholarship Council. SG reports institutional grants from Astra-Zeneca, Daiichi-Sankyo, Eli Lilly, Menarini Stemline, MSD, Novartis, Pfizer, Seagan, and Gilead. JT reports honoraria from Medtalks and CongressCars, and leadership in Medical Ethics Board Brabant, and Board of Dutch Society for Medical Oncology. KH reports institutional grants from Astra-Zeneca, Novartis, and Gilead. AF reports institutional grants and personal fees from Roche, Novartis, Pfizer, and Eli Lilly, institutional grants from Roche, Novartis, Pfizer, Eli Lilly, AstraZeneca, Daiichi Sankyo and Gilead. VTH reports institutional grants and personal fees from Roche, Novartis, Pfizer, and Eli Lilly, institutional grants from Roche, Novartis, Pfizer, Eli Lilly, AstraZeneca, Daiichi Sankyo and Gilead. MB reports institutional grants from Astra-Zeneca, Daiichi-Sankyo, Eli Lilly, Novartis, Roche, Pfizer, and Gilead, and personal consulting fees from Novartis and Pfizer. All remaining authors have declared no conflicts of interest. If there are other authors, they declare that they have no known competing financial interests or personal relationships that could have appeared to influence the work reported in this paper.

## Data Availability

The datasets generated during and/or analysed during the current study are not publicly available but are available from the corresponding author on reasonable request.
