# Supplementary material for: Complete responders in patients with HER2-positive metastatic breast cancer: a real-world SONABRE study
Source: Breast. 2025 Sep 23;84:104583. doi: 10.1016/j.breast.2025.104583 (PMC12510007; doi:10.1016/j.breast.2025.104583)

**Supplementary data for “Complete responders in patients with HER2-positive metastatic breast cancer: a real-world SONABRE study”**

**Supplementary Table 1.** Best response on first-line pertuzumab, trastuzumab plus chemotherapy of 244 patients with HER2-positive metastatic breast cancer, N (%)

| **Objective response** |  | **207 (85)** |
| --- | --- | --- |
|  | Complete response | 63 (26) |
|  | Partial response | 144 (59) |
| **Stable disease** |  | 28 (11) |
| **Progressive disease** |  | 9 (4) |

N= number of patients

**Supplementary Figure 1.** Flowchart of patients reaching complete response following first-line pertuzumab, trastuzumab, and chemotherapy


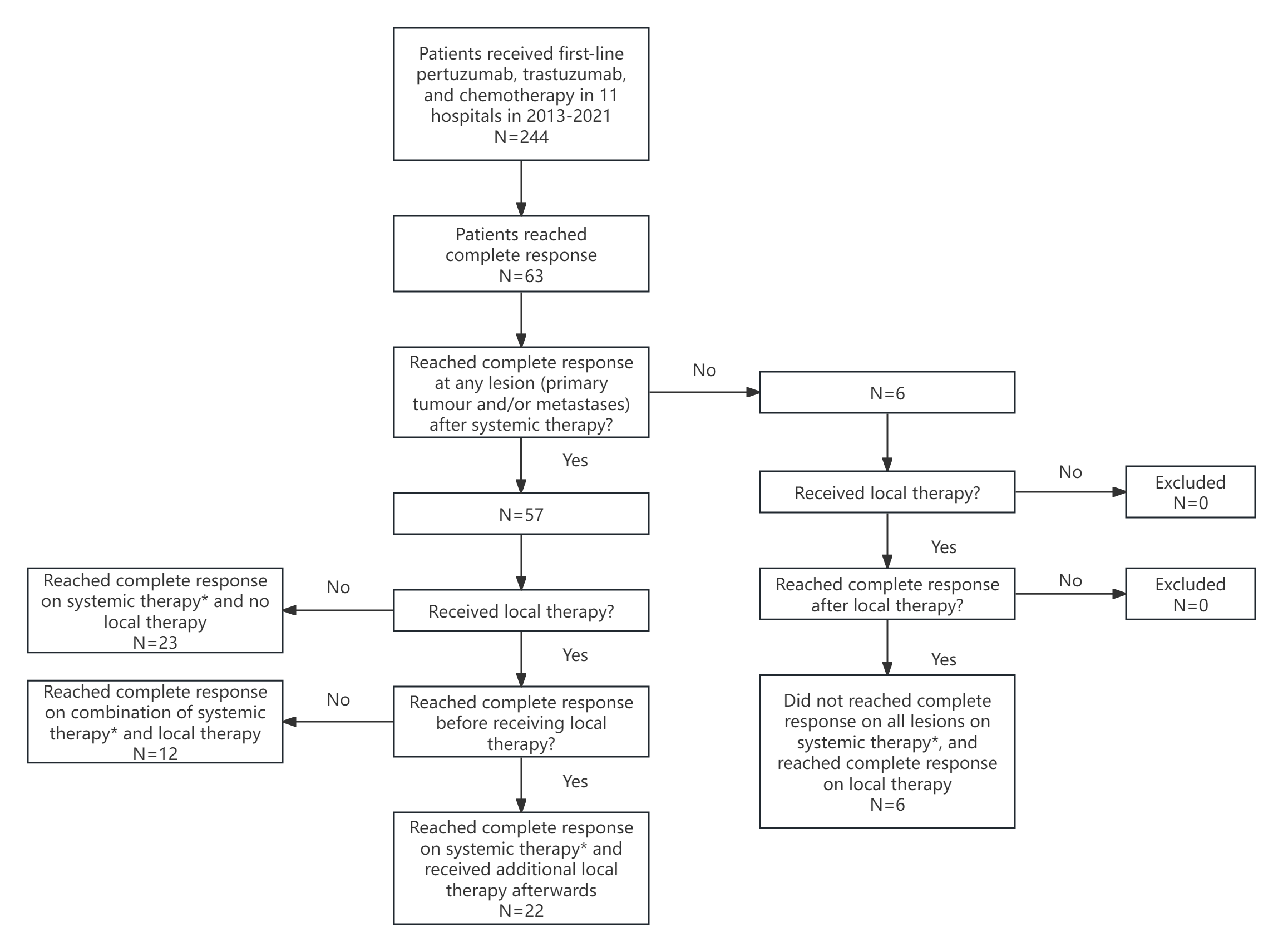


*first-line pertuzumab, trastuzumab, and chemotherapy

**Supplementary Text 1.** Examples of patients reaching complete response in different treatment pathway

Among the 63 patients who achieved a complete response (CR), 57 (90%) reached CR at any lesion (primary tumour and/or metastases) following systemic therapy. Supplementary Figure 1 outlines the different treatment pathways for these patients. Below are illustrative examples from each subgroup:

Systemic therapy only (N = 23):

Example: A patient with liver and lymph node metastases reached CR of all lesions after pertuzumab, trastuzumab, and chemotherapy alone, without receiving any local therapy.

Systemic therapy + concurrent local therapy (N = 12):

Example: A patient was diagnosed with non-metastatic breast cancer and the primary tumour was treated with right-sided mastectomy with breast reconstruction, followed by chemotherapy and trastuzumab. During the course of treatment, brain metastases were diagnosed and treated with Gamma Knife radiotherapy. Trastuzumab was continued for 17 months, after which it was discontinued. The patient remained under surveillance until the detection of liver metastases and progression of the brain metastases. The patient then received pertuzumab, trastuzumab, and docetaxel, and achieved CR following this combination of systemic therapy and local treatments.

Systemic therapy followed by local therapy (N = 22):

Example: A patient with *de Novo* metastatic breast cancer involving the primary tumour and bone metastases received six cycles of first-line pertuzumab, trastuzumab, and docetaxel, and achieved a complete response on imaging. The patient subsequently received surgery and radiotherapy of the primary tumour, as well as radiotherapy for the bone metastases.

CR achieved only after local therapy (N = 6):

Example: A patient with *de Novo* disease involving the primary tumour and liver metastases received five cycles of first-line pertuzumab, trastuzumab, and docetaxel. Imaging showed a partial response in both the liver and primary tumour. The patient subsequently received surgery and radiotherapy of the primary tumour, as well as radiotherapy for the liver metastases, and achieved a complete response afterwards.

**Supplementary Figure 2.** PFS (A) and OS (B) from the start of first-line pertuzumab, trastuzumab plus chemotherapy in all patients
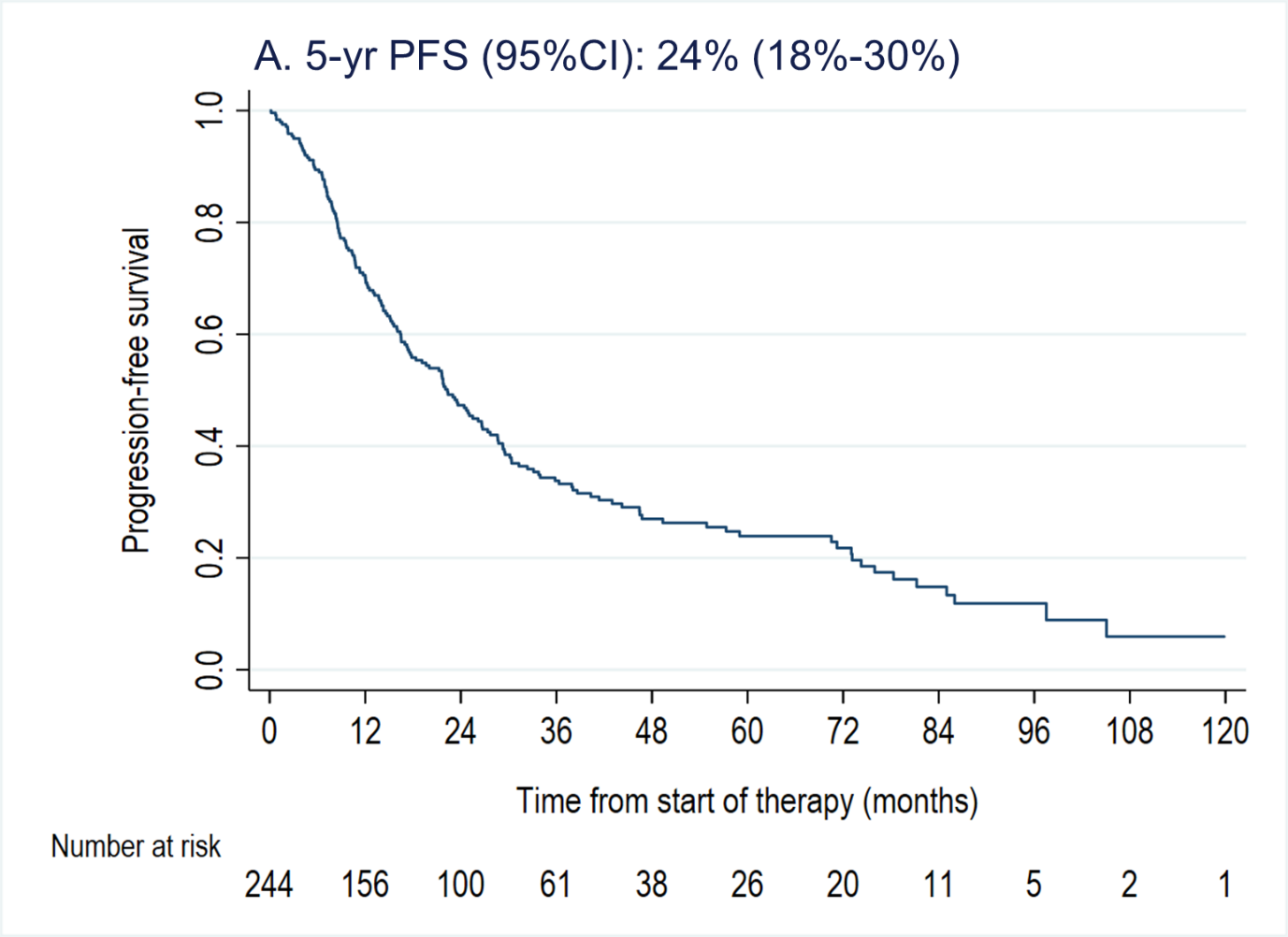

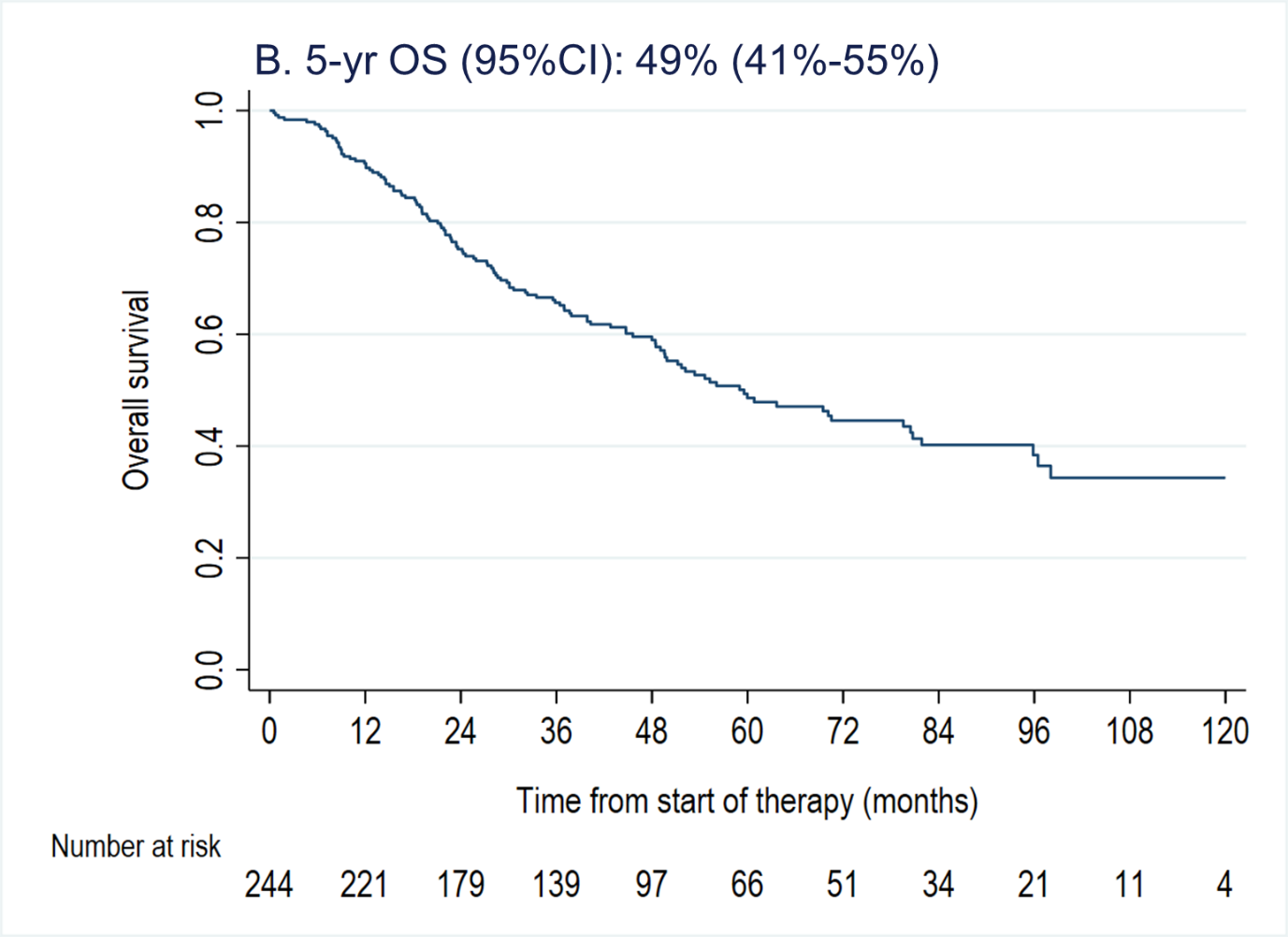

Supplement: Multimedia component 1 [file mmc1.docx]
